# An Analysis of Patient-Reported Recovery Outcomes of Topical Tripeptide/Hexapeptide Formulations Utilized in a Prospective Randomized Double-Blind Split Neck and Body Study

**DOI:** 10.1093/asjof/ojaa052

**Published:** 2020-11-18

**Authors:** Laurie A Casas, Michaela Bell, Brannon Claytor, Mary E Ziegler, Alan D Widgerow

## Abstract

**Background:**

Physicians strive to improve the postsurgical experience and optimize patient-reported recovery outcome measures (PROMs) following elective cosmetic surgical procedures. Our previous pilot feasibility study demonstrated that twice daily postoperative topical body treatment with tripeptide and hexapeptide (TransFORM Body Treatment with TriHex Technology [TFB, Alastin Skincare, Inc., Carlsbad, CA]) reduced PROMs of swelling, induration, soft tissue fibrosis, and pain as well as improved visible and palpable skin quality.

**Objectives:**

Evaluate whether adding a tripeptide/hexapeptide anhydrous gel (Regenerating Skin Nectar with TriHex Technology [RSN, Alastin Skincare, Inc., Carlsbad, CA]) pre- and post-procedure to the existing postsurgical regimen of TFB significantly improves 6 PROMs in patients undergoing neck and body contouring cosmetic surgical procedures.

**Methods:**

Ten female patients underwent 15 neck and body contouring procedures and were blindly randomized to 1 of 2 topical treatment protocols (1 [TFB] and 2 [RSN/TFB]) pre- and post-procedure. Patient-reported scores of 5 skin parameters (skin discoloration, ecchymosis, edema, induration, and subcutaneous fibrous banding) and pain scores using the Visual Analog Scale were collected at 8 intervals for 12 weeks post-procedure.

**Results:**

The treatment side that used both topicals showed significantly reduced scores of edema, induration, and subcutaneous fibrous banding compared with the side that only used 1 topical, on days 5–7 and 10–14 (*P* < 0.05). All patients observed slower soft tissue recovery on the side that was treated with TFB alone and opted to break the code and use both topical treatments.

**Conclusions:**

Patients had statistically significant improved patient-reported measures of skin edema, skin induration, and subcutaneous banding on the operated side that used both topicals.

**Level of Evidence: 2:**

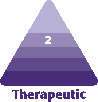

As aesthetic surgeons who perform elective cosmetic procedures, we strive to optimize the patient experience and surgical outcomes. Our previous pilot feasibility study demonstrated that twice daily postoperative topical body treatment with tripeptide and hexapeptide (TFB) compared with the control group reduced patient-reported recovery outcome measures (PROMs) of swelling, induration, soft tissue fibrosis, and pain. Improved visible and palpable skin quality was also observed.^1^ Since this publication, a clinical split-body study in patients undergoing medial thigh liposuction, demonstrated that pre- and post-procedure topical treatment with both tripeptide/hexapeptide anhydrous gel (RSN) and TFB accelerated the healing response. This conclusion was supported by results from gene expression, ultrasound, fibrometer analyses, and histology of skin biopsies that demonstrated extracellular remodeling and histological evidence of improved collagenesis and elastogenesis.^2^ The results of this recently published gene expression study demonstrated the molecular evidence for the clinical observation of accelerated post-procedure healing when participants used topical treatment with RSN and TFB,^3^ pre- and post-procedure. Other clinical studies have shown that wound bed preparation using topical RSN pre- and post-procedure accelerates healing from laser injury and provides evidence of extracellular matrix remodeling when used as preconditioning before aesthetic surgical procedures.^4,5^ TFB has also shown histologic evidence of remodeled extracellular matrix with regenerated collagen and elastin. The hexapeptide-11 component of TFB has been demonstrated to accelerate (upregulate) the process of autophagy, encouraging lipid droplet breakdown, and in vitro modeling shows macrophage recruitment to damaged fat cells with clinical trials confirming increased and hastened fat volume reduction.^1,6^

The recent gene expression study demonstrated that pre- and post-procedural topical treatment with RSN and TFB stimulated extracellular remodeling and induced anti-inflammatory genes, leading to less post-procedural induration documented by ultrasound. The question that still remains is whether both products are necessary to improve PROMs and ensure patient compliance to use 2 separate products. This split neck and body, randomized, double-blinded study was designed to analyze whether adding a second topical treatment RSN (topical treatment 2) pre- and post-procedure improved 6 PROMs or whether a single topical treatment with TFB (topical treatment 1) would achieve the same improved post-procedural recovery.

## METHODS

This split-body, randomized, double-blinded study included 10 patients, who were already scheduled for surgery between January 16, 2019, and May 06, 2019. The study concluded on August 06, 2019, after the 3-month follow-up of the 10th patient. Inclusion criteria involved patients of any age already scheduled for a neck or body contouring surgical procedure(s) involving fat reduction. Patients who met these criteria were not excluded. These 10 patients underwent 15 bilateral procedures: neck (*N* = 1) and body contouring (*N* = 14: abdominoplasty, *N* = 5; breast reduction, *N* = 1; and laser liposuction *N* = 8) (Table 1).

**Table 1.** Patient Demographics and Procedures

Patient	F/M	Age	BMI	Fitz Skin Type	Breast Reduction Bilateral	Abdominoplasty	Liposuction	Laser Liposuction
1	F	56	21.79	3	250 g per side	N/A	Extensive axilla and upper abdomen	N/A
2	F	53	28.87	4	N/A	Umbilicoplasty excision skin	Extensive upper abdomen flanks	N/A
3	F	47	35.51	2	N/A	No umbilicoplastySkin excision	N/A	N/A
4	F	55	25.06	2	N/A	N/A	N/A	Extensive arms and axilla
5	F	37	25.68	2	N/A	UmbilicoplastyExcision skin	Extensive upper abdomen and flanks	Moderate arms
6	F	41	19.13	1	N/A	UmbilicoplastyExcision skin	Minimal flanks	N/A
7	F	55	38.29	2	N/A	UmbilicoplastyExcision skin	Moderate upper abdomen and flanks	N/A
8	F	47	24.63	2	N/A	N/A	N/A	Extensive circumferential thighs R 1750 mL L 1800 mL
9	F	47	22.14	3	N/A	N/A	N/A	Minimal neck
10	F	37	27.19	2	N/A	N/A	N/A	Extensive circumferential thighs R 2500 mL L 2600 mL

BMI, body mass index.

All 10 patients were provided 2 bottles labeled 1, which contained TransFORM Body Treatment with TriHex Technology (TFB) 6 oz, and 2, which contained Regenerating Skin Nectar with TriHex Technology (RSN) 4 oz. Each patient was instructed to use the bottle labeled topical treatment 1 (TFB) on both right and left sides of their neck or body to encompass the surgical site, for 10–14 days pre-procedure and for 12 weeks post-procedure. The 10 patients were also asked to select an unlabeled envelope out of a box that contained their randomized side (right side or left side). They were instructed to treat the side written in the envelope with topical treatment 2 (RSN) twice daily pre-procedure for 10–14 days and post-procedure for 12 weeks. Abdominoplasty patients were asked to treat abdominal and flank skin except for the skin that was to be excised. This planned skin excision area was drawn out at their preoperative visit to clarify skin that was not to be treated. Post-procedure, patients were instructed to use the topical in the same surgical areas and to include the skin at the incision. Application instructions per procedure are included in Appendix 1. On the day of the surgical procedure, each patient was asked whether they had been compliant with their pre-procedure treatment regimens.

Patients were evaluated at 8 postoperative intervals: days 1–3, 5–7, 10–14, 21–25, 28–30, 35–40 and 42–50, and again at 12 weeks. At each post-procedure visit, the Visual Analog Scale (VAS) as a patient-reported outcome measure for pain was administered in person to the patient by the same medical assistant, and their score sheet for the right and the left side was placed into the patient’s study chart. The same medical assistant also asked each patient to score 5 skin parameters—ecchymosis, swelling, skin discoloration, induration, and subcutaneous banding of full stretch, for their right and left side, using a 0–4 scale (0—none; 1—barely perceptible, visually or palpably; 2—mild; 3—moderate; and 4—severe). The medical assistant provided definitions of each skin parameter. Patients wrote down a score for the right and left sides for each of the 5 skin parameters. Score sheets for every visit were kept in each patient’s study chart. The study patients were deidentified and labeled 1–10, and their data sheets were entered onto a spreadsheet by an independent data entry person. At every post-procedure visit, each patient was given the option to proceed with the study, opt out, or break the code to use topical treatment 2 (RSN) on the contralateral side that they were treating with only topical treatment 1 (TFB). This study was conducted in accordance with the World Medical Association Declaration of Helsinki’s statement of ethical principles for medical research involving human patients, including research on identifiable human material and data. Full informed written consent was acquired from each patient in the study. 

## RESULTS

Ten females (age range 37-56, mean 47.5; BMI range 19.13-38.29, mean 26.83; Fitzpatrick skin type 1-4) completed the study. The following statistical analysis was performed: 15 procedures were analyzed computing the difference in skin parameters between TFB plus RSN vs TFB alone each day. Both parametric test (ie, *t* test) and nonparametric test (ie, signed rank test) were used. Treatments were compared before and after the code was broken, that is, when patients opted to use both products. Skin parameters tested–skin discoloration, ecchymosis, edema, induration, and subcutaneous fibrous banding and pain (VAS).

All patients were compliant with their 8 postoperative visits (postoperative intervals: days 1-3, 5-7, 10-14, 21-25, 28-30, 35-40 and 42-50, and again at 12 weeks) in which a scale was used to score 6 PROMs of their right and left sides. All patients requested to break the code because they encountered a difference in PROM from the side on which they used 2 topical treatments, topical treatment 1 (TFB) and topical treatment 2 (RSN). By day 14, all patients asked to use topical treatment 2 (RSN) on both sides, except patient 10, who asked to break code on day 26 to use topical treatment 2 (RSN).

**Figure 1.**
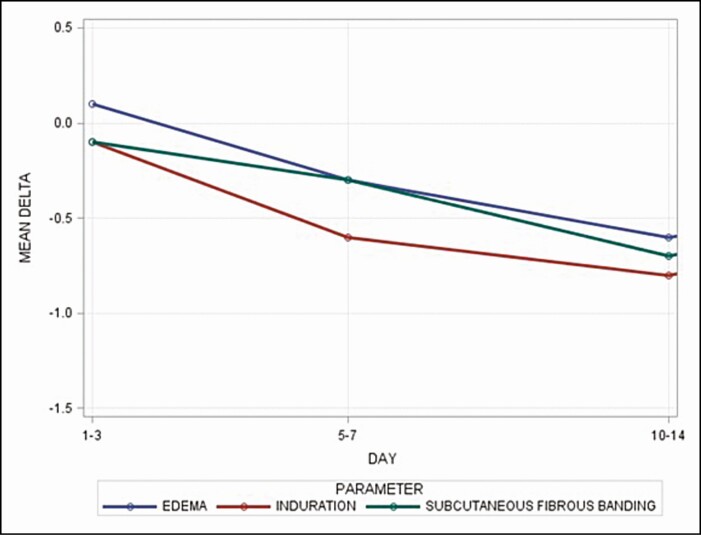
Significant reductions in edema, induration, and subcutaneous fibrous banding due to topical treatment 1 (TFB) plus topical treatment 2 (RSN) compared with topical treatment 1 (TFB) alone.

Table 2 and Figure 1 present the mean delta, standard deviation, and *P* value of the statistical tests. The results show that topical treatment 1 (TFB) plus topical treatment 2 (RSN) significantly reduced edema, induration, and subcutaneous fibrous banding compared with topical treatment 1 (TFB) alone on days 5–7 and days 10–14 (*P* < 0.05), before the code was broken, whereas reductions in skin discoloration, ecchymosis, and pain were not statistically significant. Please note that the results were similar when the data on broke days were excluded from the analysis (data not shown).

**Table 2.** Comparison of Skin Parameters Between Topical Treatment 1 (TFB) Plus Topical Treatment 2 (RSN) vs Topical Treatment 1 (TFB) Alone (Before and on Broke Day, Topical Treatment 1)

DAY	Parameter	Mean Delta	SD	Min	Max	*P*-value
Days 1-3	Skin discoloration	−0.1	0.5	−1	1	0.5816
	Ecchymosis	−0.3	0.5	−1	0	0.0406
	Edema	0.1	0.3	0	1	0.3343
	Induration	−0.1	0.4	−1	0	0.1648
	Subcutaneous fibrous banding	−0.1	0.3	−1	0	0.3356
	Pain (VAS)	−0.4	1.7	−6	2	0.3840
Days 5-7	Skin discoloration	−0.2	0.4	−1	0	0.0824
	Ecchymosis	−0.1	0.6	−1	1	0.4332
	Edema	−0.3	0.5	−1	0	0.0406
	Induration	−0.6	0.5	−1	0	0.0003
	Subcutaneous fibrous banding	−0.3	0.5	−1	0	0.0401
	Pain (VAS)	−1.0	1.9	−5	1	0.0552
Days 10-14	Skin discoloration	−0.1	0.3	−1	0	0.3356
	Ecchymosis	−0.4	0.8	−2	1	0.1365
	Edema	−0.6	0.6	−2	0	0.0057
	Induration	−0.8	0.7	−2	0	0.0008
	Subcutaneous fibrous banding	−0.7	0.6	−2	0	0.0019
	Pain (VAS)	−0.6	1.3	−4	1	0.1197

VAS, Visual Analog Scale.

**Figure 2.**
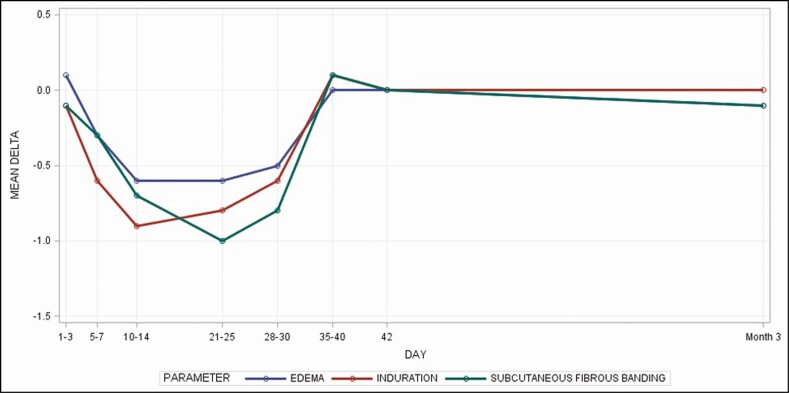
This figure demonstrates that skin parameters treated with topical treatment 1 (TFB) plus topical treatment 2 (RSN) as compared to topical treatment 1 (TFB) alone decreased before the broke days, increased after the broke days, and settled at zero in month 3.

Figure 2 shows that skin parameters treated with topical treatment 1 (TFB) plus topical treatment 2 (RSN) as compared with topical treatment 1 (TFB) alone decreased before the broke days, increased after the broke days, and settled at zero in month 3.

**Figure 3.**
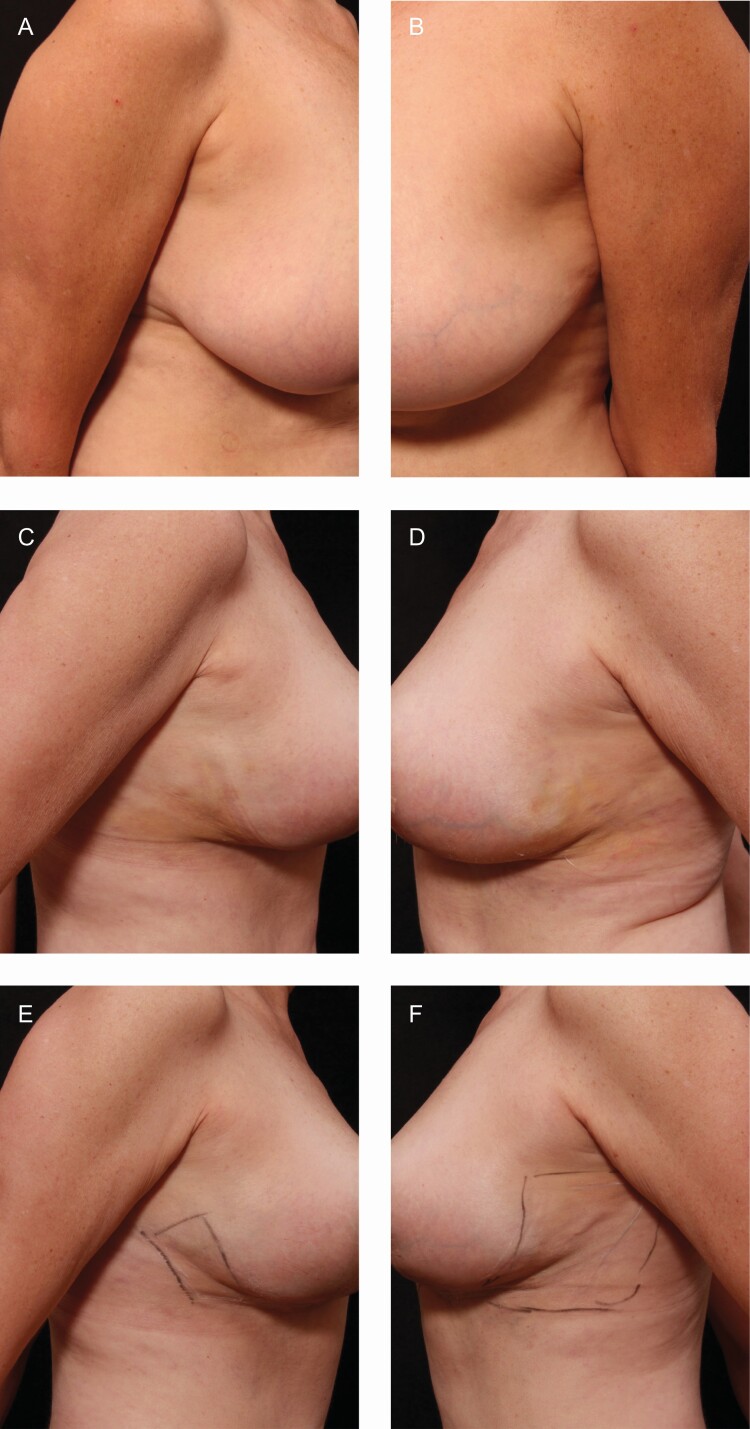
Patient 1—procedure: breast reduction and axillary liposuction. The 56-year-old female patient used both TFB and RSN on the right side. (A, B) Preoperative right and left view, (C, D) 2 weeks postoperative right and left view, and (E, F) 4 weeks postoperative right and left view with marking. Marking encompasses the area the patient felt edema/induration and subcutaneous fibrosis.

**Figure 4.**
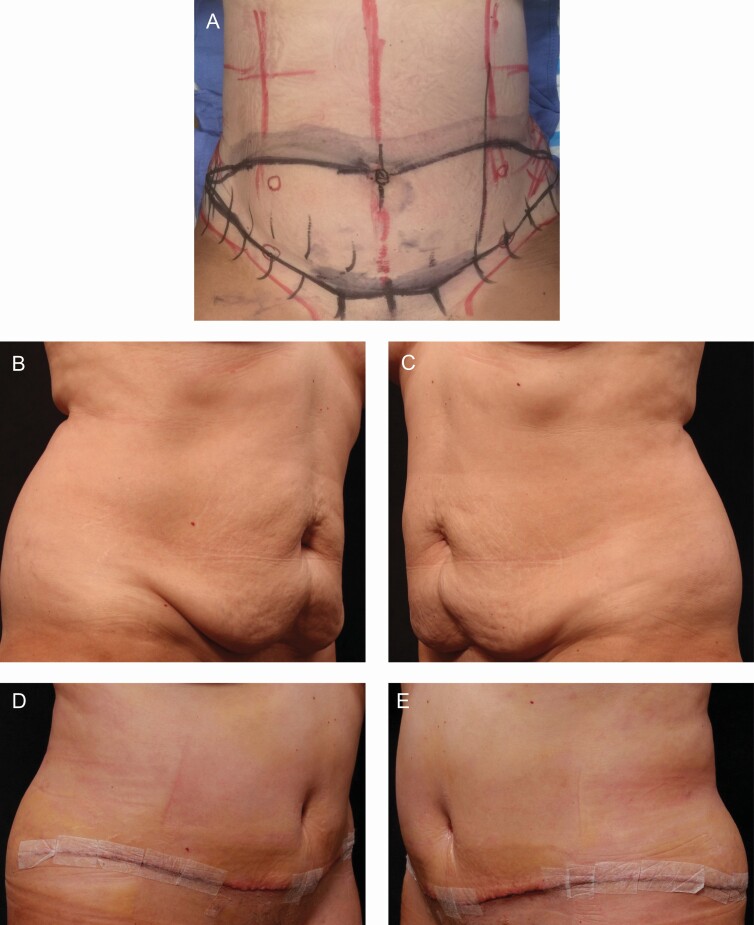

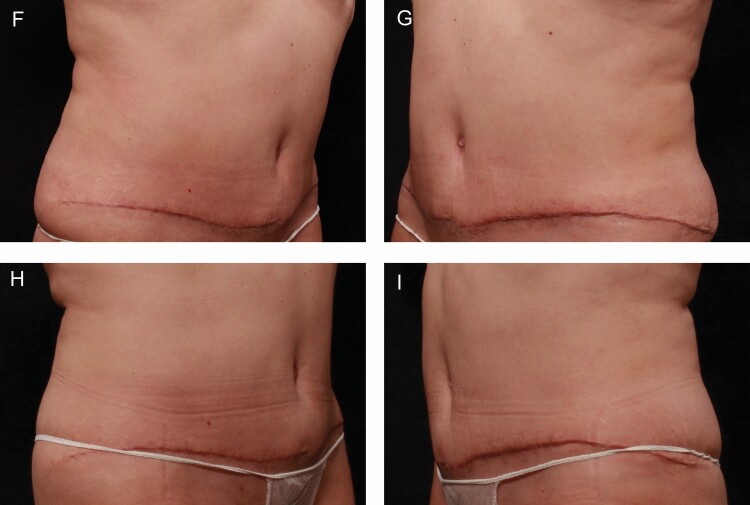
Patient 2—procedure: abdominoplasty. The 53-year-old female patient used both TFB and RSN on the right side. (A) Preoperative markings view, (B, C) preoperative right and left view, (D, E) 1 week postoperative right and left view, (F, G) 3 weeks postoperative right and left view, and (H, I) 5 weeks postoperative right and left view.

Figures 3 and 4 represent two patient photographs; being that this was a patient-reported outcome study and both sides were treated with at least 1 topical product, differences in the 2 sides were difficult to capture within a photograph.

## DISCUSSION

The basis of the topical combination therapy devised in these cases relates to the advantages reported by preconditioning the skin before surgical procedures,^5,7,8^ together with the concept of the clearance of “waste products” related to surgery or metabolism, in this case, lipid droplets. It is hypothesized that lipid droplets create localized areas of inflammation, inflammasomes, which are not only large particles for macrophage digestion but also elaborate inflammatory mediators, which manifest as edema, induration, and in some cases localized fat necrosis. By preparing and remodeling the extracellular matrix before surgery^7,8^ and optimizing macrophage phagocytosis of lipid droplets,^6,9^ the clearance provided translates into improved skin induration and edema and lessened patient discomfort.

The 10 patients who underwent neck and body contouring procedures had statistically significantly improved patient-reported measures of skin edema, skin induration, and subcutaneous banding in the operated areas that underwent trauma from laser energy, mechanical liposuction, and skin undermining when they used the combination of topical treatment 1 (TFB) and topical treatment 2 (RSN) pre- and post-procedure. The skin parameters assessed that were treated with TFB and RSN as compared with TFB alone decreased before the code was broken, increased after the broken code, and settled at zero at month 3. This translated to a statistically significant decrease in edema, induration, and fibrous banding in the combination group up to the days that patients opted to use both products. This difference then dissipated (increased after broken code) and normalized at month 3. Specifically from a physiological perspective, the first 5–7 days would traditionally be the “clearance” phase of inflammation where proinflammatory particles are removed, and 10–14 days would be the inflammatory “switch off” phase where extracellular remodeling is producing new collagen and elastin. This would translate to a maximal decrease in induration and edema in the first 5–7 days with stabilization of the process and early fibrous banding at 10–14 days. These findings are consistent with the theory proposed above and with published gene marker and gene expression studies.^3^

While this provides validation for the use of the 2 products in early recovery (14 days post-procedure), the broken codes do create limitations in analyzing the long-term impact of the use of the products. That noted, an additional liposuction study does present long-term improved outcomes with the use of these products in a split-body study.^2^ Additional limitations include smaller sample size and lack of a control group that could have used 2 bland topicals.

## CONCLUSIONS

The 10 patients who underwent 15 neck and body contouring procedures that created trauma from laser energy, mechanical liposuction, and skin undermining had statistically significantly improved patient-reported outcome measures of skin edema, skin induration, and subcutaneous banding on the operated side that used the combination of topical treatments 1 (TFB) and 2 (RSN) 10–14 days pre-procedure and 12 weeks post-procedure. The data from the PROMs from this study support the use of combination treatment with both TFB and RSN to improve PROMs and their post-procedural experience.

## Supplementary Material

ojaa052_suppl_Supplementary_Appendix_1
