# Supplementary material for: An Analysis of Patient-Reported Recovery Outcomes of Topical Tripeptide/Hexapeptide Formulations Utilized in a Prospective Randomized Double-Blind Split Neck and Body Study
Source: Aesthet Surg J Open Forum. 2020 Nov 18;3(1):ojaa052. doi: 10.1093/asjof/ojaa052 (PMC7984834; doi:10.1093/asjof/ojaa052)
Supplement: ojaa052_suppl_Supplementary_Appendix_1 [file ojaa052_suppl_supplementary_appendix_1.docx]

**APPENDIX 1:**

**Score Sheet Forms in Study Packet**

**Labelled Patient #1-10 _________________Procedure #_____**

**Date ______ Weight ______ (Preop Weight________)**

**Right    Left**

0-4 scale for scoring

0-none

1-barely perceptible

2-mild

3-moderate

4-severe

Patient Instructed to compare skin to non-operated area-

Score Sheet **Right** side and **Left**Side

1. Ecchymosis (Colors of a typical bruise, pictures of (0-4)
2. Swelling (Compared to non-operated body skin, body weight on the scale (0-4)
3. Skin Discoloration (Red, pigmentation changes (0-4)
4. Induration (def. area of hardness in the skin, skin thickness and firmness, (0-4)
5. Subcutaneous banding (evaluated on full stretch e.g. the patient fully extended her arms or neck and did a side bend and back bend. The patient noted severity of the tightness and resistance from pulling internally when attempting these maneuvers and the palpable fibrous banding) scored (0-4)

**Liposuction and Degree of Liposuction Performed-defined by cc Removed**

                                                                   Minimal             <100      cc

                                                                   Moderate          100-500 cc

                                                                   Extensive          >500 cc

**Type of Liposuction**

1. Traditional 3mm and/or 4mm canula
2. Laser 1064/1440 fibers

**Appendix:**

Application of each of the products were as follows:

TFB to both sides and RSN to the designated randomized side.

2 pumps per side for the abdominoplasty (preop exclude skin to be excised)

2 pumps per leg for lipo leg cases

1 pump per side for arm cases

1 pump per side for axilla lipo cases

1 pump per side for hip lipo cases

½ pump per side for neck cases

Approximately 700 pumps in TFB (6oz) and 500 pumps in RSN (4oz)

Prior to procedure patients were instructed to pump the amounts according to their procedure and apply to the surgical area. Abdominoplasty patients were asked to treat abdominal and flank skin except for the skin that was to be excised. This planned skin excision area was drawn out at their preoperative visit to clarify skin that was not to be treated. Post procedure patients were instructed to use the topical in same surgical areas and to include the skin at the incision.
